# Distribution of the Condylion-Gonion-Menton (CoGoMe^) Angle in a Population of Patients from Southern Italy

**DOI:** 10.3390/dj7040104

**Published:** 2019-11-03

**Authors:** Vincenzo D’Antò, Ada Carolina Pango Madariaga, Roberto Rongo, Rosaria Bucci, Vittorio Simeon, Lorenzo Franchi, Rosa Valletta

**Affiliations:** 1 Department of Neurosciences, Reproductive Sciences and Oral Sciences, Section of Orthodontics, University of Naples “Federico II”, 80131 Naples, Italy; vincenzo.danto@unina.it (V.D.); apangom@gmail.com (A.C.P.M.); rosaria.bucci@unina.it (R.B.); valletta@unina.it (R.V.); 2 Department of Mental Health and Preventive Medicine, Medical Statistics Unit, University of Campania “Luigi Vanvitelli”, 80138 Naples, Italy; vittoriosimeon@gmail.com; 3 Department of Experimental and Clinical Medicine, Section of Dentistry, Orthodontics, University of Florence, 50134 Florence, Italy; lorenzo.franchi@unifi.it; 4 Department of Orthodontics and Pediatric Dentistry, School of Dentistry, The University of Michigan, Ann Arbor, MI 48109, USA

**Keywords:** craniofacial growth, cephalometric analysis, digital orthodontics, sagittal jaw relationship, mandibular divergence, orthodontic treatment planning

## Abstract

The condylion-gonion-menton angle (CoGoMe^) is commonly used as a pre-treatment indicator of responsiveness in Class II patients treated with functional appliances. The distribution of this angle in the Caucasian population is still unknown. This study aimed to determine the distribution of the CoGoMe^ and its relationship with age, sagittal jaw relationship (ANPg^), and mandibular inclination (SN^GoGn) in patients from Southern Italy. The sample included 290 subjects (median14 years of age; Interquartile range, IQR, 12–17) with lateral cephalograms taken before the orthodontic treatment. The distribution of the CoGoMe^ was assessed with the Shapiro–Wilk test, and the differences according to the ANPg^ and the SN^GoGn were estimated using one-way ANOVA. Linear regression analysis was performed to evaluate how the CoGoMe^ varied according to age. The statistical significance was set at P < 0.05. The results showed that the CoGoMe^ was normally distributed (P = 0.290) with a mean value of 127.2° ± 7.7°. The distribution of the CoGoMe^ in groups with different SN^GoGn angles was significantly different (P < 0.001). These angles showed a positive association (Beta coefficient B = 0.6; 95% CI: 0.51, 0.67; P < 0.001). In growing patients, the CoGoMe^ decreased every year by 0.6° (B = −0.6; 95% CI: −1.05, −0.12; P = 0.014). In conclusion, the CoGoMe^ was associated with mandibular inclination and could be considered to be a predictor of vertical growth patterns.

## 1. Introduction

The purpose of orthodontic treatment is to achieve an aesthetic improvement and provide functional occlusion and balanced facial features [1]. A precise diagnosis is essential for choosing the correct therapy and determining the prognosis adequately. Therefore, orthodontic treatment planning requires an accurate prediction of the amount and direction of craniofacial development [2,3,4]. Since Broadbent [5] introduced lateral cephalometric radiography in 1931, studies on craniofacial growth and development have increased in number and many researchers have suggested definitions and norms for the normal occlusion. Hence, radiographic cephalometry has become one of the most important instruments of clinical and research orthodontics [6].

The appropriate interpretation of any cephalometric analysis requires norms that are calculated from populations and adjusted according to age, gender, and ethnic group [5,6]. The cephalometric value norms represent a valuable aid for clinicians to determine the measure of deviations from the population average, or what is considered “healthy”. Currently, orthodontic patients in clinical practice range from children to adults and they belong to a variety of ethnic groups; therefore, a wide range of representative standards would ideally be needed to perform an individualised orthodontic treatment plan [7,8].

Mandibular growth prediction is a factor of utmost importance in orthodontic/orthopaedic treatment planning [9]. Indeed, it seems crucially important to identify the mandibular growth pattern before treatment, as patients with signs of posterior mandibular growth rotation (hyperdivergent growth pattern) are assumed to be more difficult to treat than those with an anterior mandibular rotation (hypodivergent growth pattern) [10,11,12]. The most widely used method for establishing the jaw growth rotation is cephalometric analysis. Several different analyses have been introduced to evaluate a patient’s divergency, such as the Ricketts analysis or the Jarabak analysis [13,14].

The SN^GoGn is a very useful diagnostic parameter to consider before starting an orthodontic treatment because it evaluates the facial pattern of a subject and it reflects the variability of the mandibular plane in relation to the anterior cranial base [15].

Another important morphological characteristic of the lower jaw related to the anterior/posterior rotational growth pattern is the angle formed by the condylar axis (CoGo) and the mandibular base (GoMe), i.e., the Condylion-Gonion-Menton angle (CoGoMe^) [10,11]. Although this angle has been proposed as a possible predictor of responsiveness during orthopaedic therapies [16], there are no studies on the distribution of the CoGoMe^ and its relationship with classical cephalometric vertical (SN^GoGn) and sagittal measurements (ANPg^).

Finally, ANPg^ is an angle useful for the sagittal classification of the malocclusion, Class I, Class II, or Class III skeletal relationship, and it is formed by the NA (Nasion-point A line) line through N and A and the NPg (Nasion-Pogonion line) line through N and Pg.

Therefore, the aim of this study was to determine the distribution of the CoGoMe^ and its relationship with age, sagittal jaw relationship (ANPg^), and mandibular inclination (SN^GoGn) in a population of patients from Southern Italy. The null hypothesis was that there is no relationship between the CoGoMe^ and the SN^GoGn.

## 2. Materials and Methods

This research protocol was approved by the Ethics Committee of the University of Naples Federico II (121/19; 18 March 2019).

For this retrospective study, the lateral cephalograms of patients, treated at the Section of Orthodontics at the University of Naples Federico II, were screened. Due to the retrospective design of the study, it was not possible to obtain the informed consent from all the participants, however, before orthodontic treatment, all patients provided authorization to use their clinical records for research purposes.

The lateral cephalograms were selected based on the following inclusion criteria:

- age ≥8
- a good quality lateral x-ray

The following conditions were considered as exclusion criteria:

- patients with systemic diseases
- patients with genetic syndromes
- previous orthodontic treatment

All the lateral radiographs were taken before the orthodontic treatment in natural head position [17,18]. One operator traced all lateral cephalograms with a cephalometric software program (Dolphin, Chatsworth, CA, USA).

**Figure 1:**
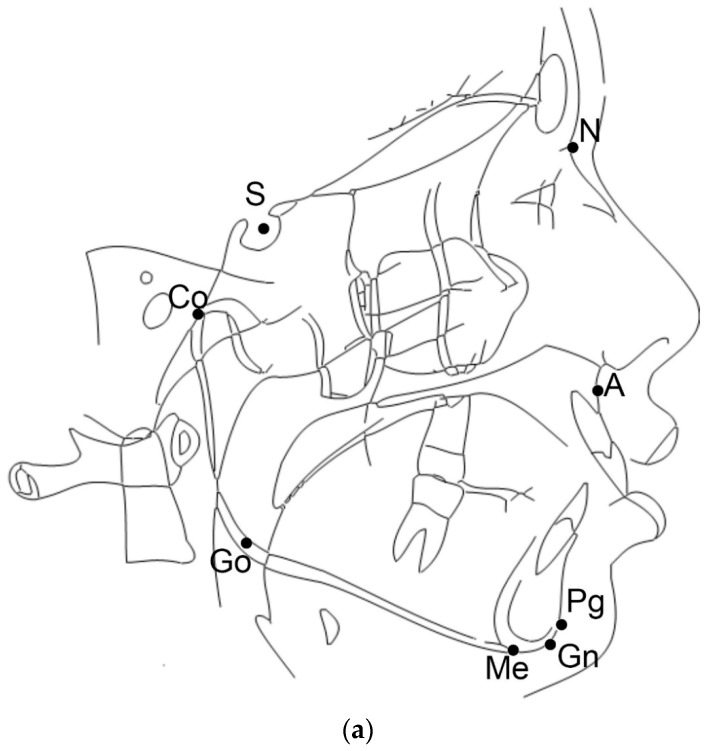

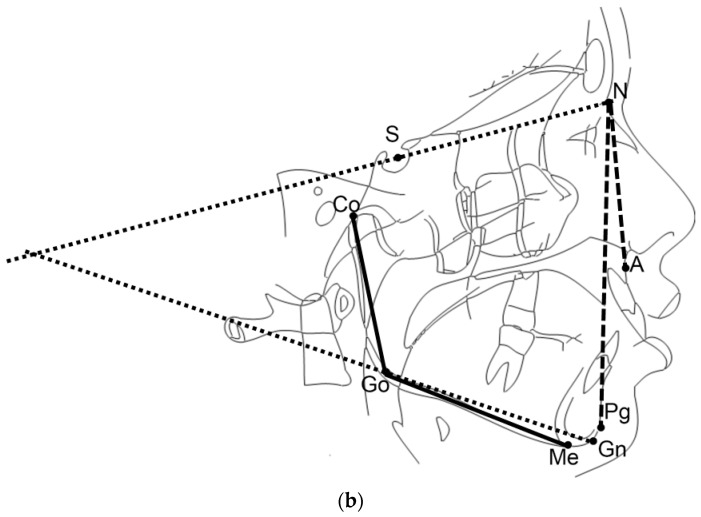
(**a**) Cephalometric analysis and landmarks. Landmarks: A (Point A), most posterior point of the frontal concavity of the maxillary between the anterior nasal spine and the alveolar processes; N (Nasion), most anterior point of the junction of the nasal and frontal bone (frontonasal suture); S (Sella), centre of the hypophyseal fossa; Go (Gonion), midpoint of the curvature at the angle of the mandible; Co (Condylion) the highest and most posterior point on the contour of the mandibular condyle; Pg (pogonion), the most anterior point of the symphysis; Gn (Anatomical gnathion), point of the mandibular symphysis on the facial axis; and Me (Menton), most inferior point of the mandibular symphysis. (**b**) Reference: NA (Nasion-point A line) line through N and A; NPg (Nasion-Pogonion line) line through N and Pg; SN (Sella-Nasion line) line through S and N; GoGn (Mandibular plane) line through Go and Gn; CoGo (condylar axis) line through Co and Go; and GoMe (Mandibular base) line through Go and Me. 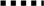 SN^GoGn, 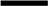 CoGoMe^, 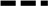 ANPg^.

For this study, the cephalometric analysis was performed as shown in Figure 1a,b. Briefly, three cephalometric variables were assessed: the CoGoMe^ measured the mandibular structure, which is the angle between the condylar axis (Condylion-Gonion) and the mandibular base (Gonion-Menton); the SN^GoGn determined jaw divergence, which is the angle between the anterior cranial base (Sella-Nasion) and the mandibular plane (Gonion-Gnathion); and the ANPg^ assessed sagittal jaw discrepancy, which is the angle between the Nasion-point A line and the Nasion-Pogonion line [19].

**Figure 2:**
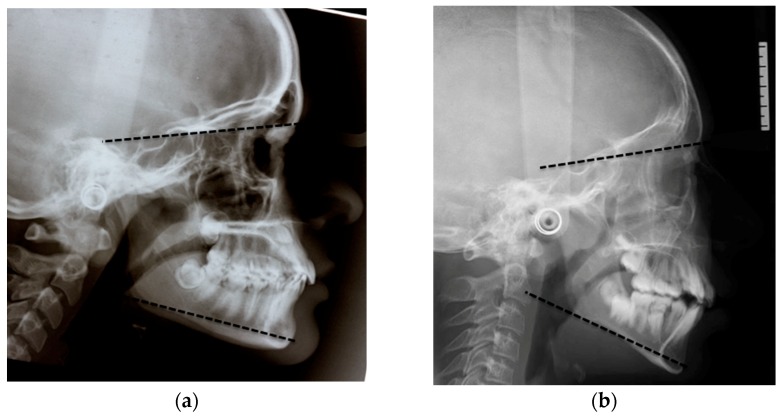

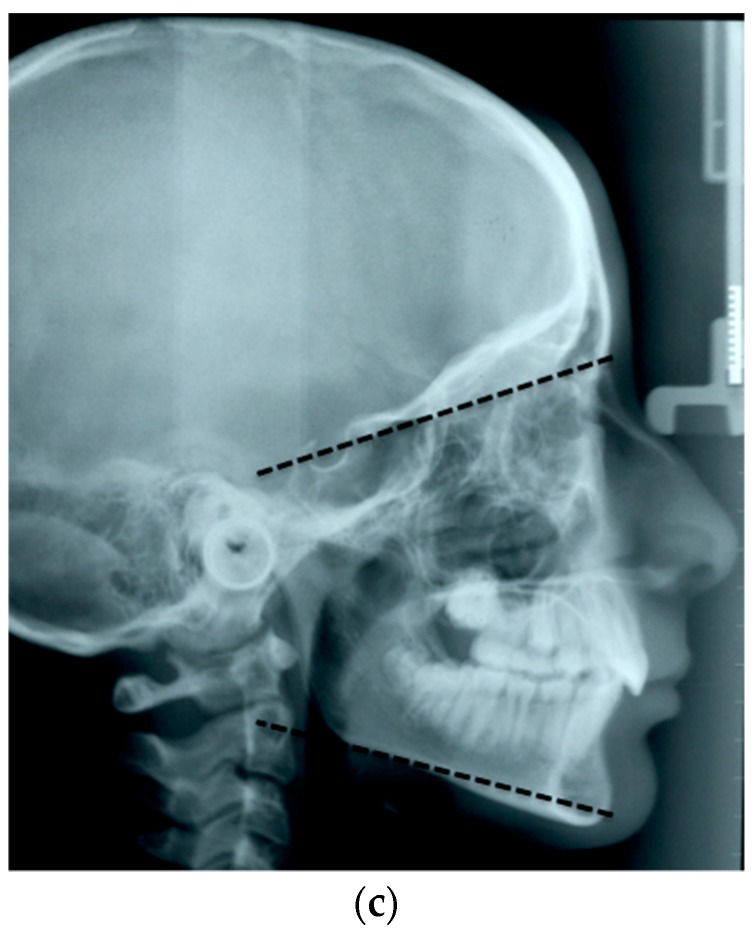
(**a**,**b**) Hypodivergent and hyperdivergent patients according to SN^GoGn; and (**c**) Normodivergent patient according to SN^GoGn.

The sagittal malocclusion was classified into three groups according to the ANPg^: Class III with an ANPg^ equal to or less than −1°, Class I with an ANPg^ between −1° and 5°, and Class II with an ANPg^ equal to or greater than 5°. Similarly, the sample was divided into three groups according to their vertical malocclusion: hypodivergent with an SN^GoGn equal to or less than 27°, normodivergent with an SN^GoGn between 27° and 37°, and hyperdivergent with a SN^GoGn equal to or greater than 37°, as seen Figure 2a–c.

### Statistical Analysis

The Dahlberg’s formula [20] and the paired Student’s t-test with the type I error set at 0.05 (P < 0.05) were used to assess the method of error. Hence, 101 randomly selected lateral cephalograms were reassessed by the same examiner after a memory washout period of at least 8 weeks. 

Categorical variables were reported as frequencies and percentages, and continuous variables were reported as means and standard deviations if the data distribution was normal or as medians and interquartile range if the data showed a skewed distribution. The Shapiro–Wilk (SW) test was used to evaluate normality assumption.

The Pearson correlation analysis was used to assess the relationship between continuous variables, when requested.

Differences in the CoGoMe^ among individuals with different ANPg^ and SN^GoGn were estimated, as appropriate, by using one-way Analysis of Variance (ANOVA).

Linear regression analysis was performed to evaluate (1) how the CoGoMe^ (used as a dependent variable) changed according to age and (2) how the CoGoMe^ (used as an independent variable and adjusted for age) was able to predict the SN^GoGn. For the first issue, two models for linear regression analysis were performed. One model included growing patients younger than 17 years of age and the other included patients aged 17 years of age and older. Beta coefficients and 95% confidence intervals were calculated.

The level of statistical significance was set at P < 0.05. Statistical analysis was performed using STATA version 14.0 (StataCorp LP, Stata Statistical Software, College Station, TX, USA).

## 3. Results

The sample included 290 subjects: 122 males (42.1%) and 168 females (57.9%), aged 8 to 53 years (median 14; Interquartile range IQR 12–17).

The method error for the three angles assessed in the study was ANPg^ = 0.4°, SN^GoGn = 0.9°, and CoGoMe^ = 1.3°, and there were no systematic errors for any measurements (P > 0.05).

**Figure 3:**
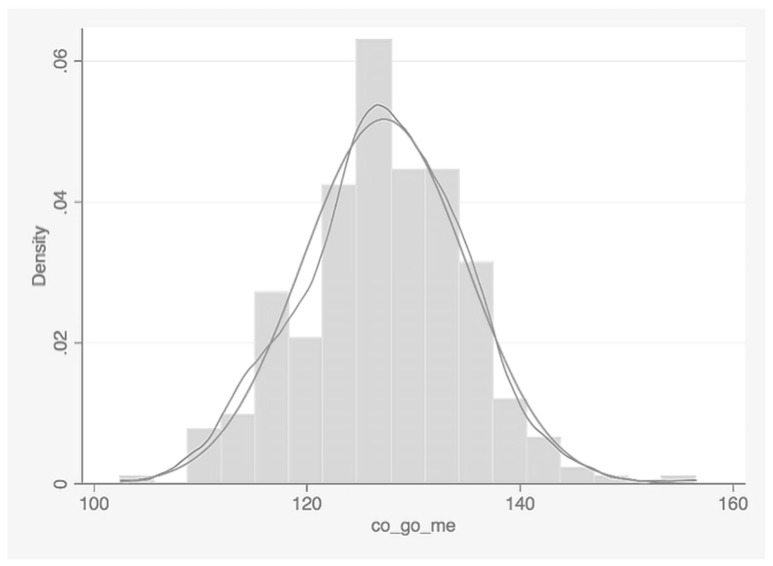
Graph describing the distribution of the CoGoMe^ in the study population (N = 290; mean ± SD = 127.2° ± 7.7° [CI 95% 112.1°–142.3°]).

In the total sample of 290 patients, the CoGoMe^ was normally distributed (SW test, P = 0.290), with a mean value of 127.2° ± 7.7°, as seen in Table 1 and Figure 3. The ANPg^ and the SNGoGn^ presented a mean value of 2.6° ± 3.2° and 31.9° ± 6.8°, respectively (Table 1).

After dividing the sample into three groups according to the ANPg^, the CoGoMe^ showed no statistically significant difference (P = 0.560). In particular, Class III (ANPg^ ≤−1°) included 32 patients and showed a mean CoGoMe^ of 128.59° ± 7.8°; Class I (−1°< ANPg^ <5°) included 196 patients and presented a mean CoGoMe^ of 127.09° ± 7.8°; and Class II (ANPg^ ≥5°) included 62 patients and showed a mean CoGoMe^ of 126.9° ± 7.2°, as seen in Table 2.

**Figure 4:**
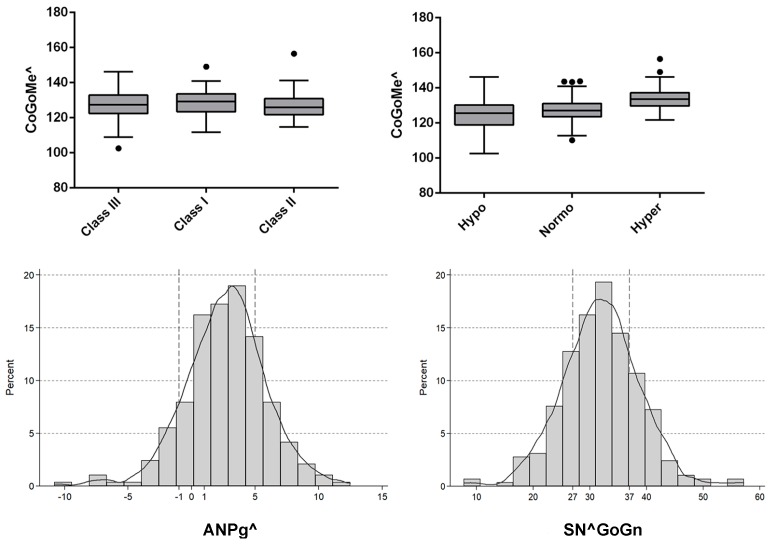
Box-and-whiskers plots (upper panel) of the CoGoMe angle by ANPg^ and SN^GoGn. Line in the box: median value. Box hinges: 25th–75th percentiles; ends of the segments: 5th–95th percentiles; and dots: outliers. Histograms with kernel distribution (lower panel) were presented to describe ANPg^ and SN^GoGn variables. Cut-off values were highlighted with dashed line (−1 and 5).

When the sample was divided into three groups according to the SN^GoGn, a statistically significant difference in the CoGoMe^ was observed (P < 0.001). In particular, 60 patients were hypodivergent (SN^GoGn <27) and presented a mean CoGoMe^ of 120.1° ± 6.63°; 166 patients were normodivergent (27≤ SN^GoGn ≤37) and presented a mean CoGoMe^ of 127.1° ± 6.11°; and 64 patients were hyperdivergent (SN^GoGn >37) and presented a mean CoGoMe^ of 134.02° ± 6.18°, as shown in Table 2 and Figure 4.

The correlation between the CoGoMe^ and the SN^GoGn was moderate (Pearson’s r, r = 0.6, P < 0.0001). On the other hand, the correlation between the CoGoMe^ and the ANPg^ was absent (r = −0.02, P = 0.74), while a weak correlation was observed between the SN^GoGn and the ANPg^ (r = 0.21, P = 0.0003).

In the linear regression analysis performed on patients under 17 years of age (N = 210), a clear decrease of the CoGoMe^ during growth was observed (beta coefficient, B = −0.6; 95% CI: −1.05, −0.12; P = 0.014), as shown in Table 3. However, in the liner regression performed on subjects older than 17 years of age (N = 80), this association disappeared and the angle remained stable over time (B = 0.004; 95% CI: −0.31, 0.32; P = 0.98), as seen in Table 3.

Finally, the results of the regression model with the SN^GoGn as the dependent variable reported that each degree of increase in the CoGoMe^ resulted in an increase in the SN^GoGn by 0.6° (B = 0.6; 95% CI: 0.51, 0.67; P < 0.001, Table 3).

## 4. Discussion

The aim of this study was to determine the distribution of the CoGoMe^ in a population of patients from Southern Italy and to assess the association of this mandibular angle with vertical and sagittal cephalometric parameters. The results showed that the CoGoMe^ was normally distributed in the studied population, and it was correlated to the vertical facial type (SN^GoGn). However, it was not influenced by the anteroposterior jaw relationship (ANPg^).

Our study is the first to report a strong association between the CoGoMe^ and the SN^GoGn, with these two angles positively correlated. Indeed, each degree of increase of the CoGoMe^ resulted in an increase of the SN^GoGn by 0.6°. Moreover, the mean value of the CoGoMe^ was statistically significantly different according to the identified subgroups of the SN^GoGn. Hence, the CoGoMe^ could help to identify mandibular growth patterns, and therefore clinicians are suggested to consider this variable carefully at the beginning of the orthodontic therapy. Indeed, CoGoMe^ might be useful to understand the mandibular rotational pattern, giving more accurate information than the SN^GoGn, that is influenced also by the inclination of the anterior cranial base [15]. The CoGoMe^ is a variable related only to mandibular structure (condylar axis and mandibular base), hence its evaluation is not affected by any other external structures. This strong correlation between CoGoMe^ and SN^GoGn is related both to an anatomical consideration—both angles evaluate the mandibular base—and to functional consideration—usually hyperdivergent patients have a lower muscles thickness and a lower bite force—that might have less control on the vertical growth pattern [21,22].

In the current study, the CoGoMe^ decreased with growth up to 17 years of age. Björk and co-workers [23,24] studied mandibular rotation and distinguished 2 types of rotation, internal and external, by superficial remodelling. From the age of 4 years to adulthood, the internal rotation is about 15° forward, while the external rotation is about 11°/12° backward, producing a 3°/4° total decrease of the mandibular angle during growth [23,24]. Hence, the natural backward rotation of the mandible during growth might be responsible for the reduction of the CoGoMe^ observed in the current study. This study included patients equal to or older than 8 years old because it is the minimum age when a lateral cephalogram is usually indicated. The age of 17 years old was considered as an average age of growth end [19,25].

The clinical significance of this study is related to the importance of growth predictors for the orthodontic diagnosis and treatment planning, with possible implications on the success rate and the duration of the orthodontic treatment for each specific malocclusion [26]. During orthodontic diagnosis and treatment planning, the possibility to correctly identify the mandibular rotational pattern during growth is a fundamental factor [16]. It is well recognised that patients with a hyperdivergent mandibular growth pattern are more difficult cases [9,10,17]. Not only the cephalometric analysis but also anatomical characteristics were used to identify the mandibular rotational patter. Already in the early 1970s, Björk and Skieller underlined the possibility of predicting the mandibular growth pattern by looking at some specific anatomic mandibular structures in longitudinal lateral cephalograms with the purpose of identifying facial morphology and the progression of mandibular rotation [23,24,25]. They introduced seven mandibular morphological signs that identified hyperdivergent and hypodivergent mandibular patterns [23]. Although, the CoGoMe^ is a cephalometric angle, it is strongly related to the mandibular anatomy and, due to its correlation with the SN^GoGn, it might improve the accuracy of the cephalometric diagnosis.

Class II malocclusion is one of the most prevalent orthodontic problems in the Caucasian population [27,28,29]. It might cause detrimental aesthetic effects and social impairment in children’s daily lives as it affects their oral-health-related quality of life, and it is a risk factor for dental traumas [30]. In growing subjects, one treatment option to correct skeletal Class II malocclusions uses functional/orthopaedic appliances, [31] but, still, great variability in the achievable mandibular advancement has been observed across the literature due to numerous factors. One factor that might be responsible for different growth potentials is mandibular morphology. Petrovic pointed out that the individual mandibular growth potential and the responsiveness to the functional orthopaedic treatment were strongly influenced by the mandibular growth pattern [10,11]. The CoGoMe^ was proposed as a pre-treatment indicator of lower jaw responsiveness in Class II patients treated with functional appliances at the mandibular growth spurt [16]. The cut-off degree of the CoGoMe^ greater or less than 125.5° was found by Franchi and Baccetti in their work of 2006 [16]. These authors suggested that the CoGoMe^ could be used for an efficient discrimination between good (CoGoMe^ <125.5°) and bad (CoGoMe^ >125.5°) responders to functional treatment of skeletal Class II malocclusion due to mandibular retrusion. This is the first study that evaluated the distribution and the associations of the CoGoMe^ with the SN^GoGn and the ANPg^ in a large population from Southern Italy, providing cephalometric norms for Caucasian patients.

The limitation of this study was that, due to ethical issues, it was not possible to collect an untreated longitudinal sample.

## 5. Conclusions

In conclusion, this study showed the following:

- In the studied sample, the CoGoMe^ presented a mean value of 127.2° ± 7.7°.
- Skeletal sagittal jaw discrepancies did not influence the CoGoMe^.
- From 8 to 17 years of age, the CoGoMe^ decreased 0.6° per year.
- For each degree of increase of the CoGoMe^, the SN^GoGn increased by 0.6°.
- The CoGoMe^ can be considered a useful cephalometric parameter for the diagnosis of the vertical facial growth pattern.

## Figures and Tables

**Table 1:** Cephalometric values in the study sample.

Variables	Mean	Median	Standard Deviation
ANPg^	2.6°	2.9°	3.2°
SN^GoGn	31.9°	32°	6.8°
CoGoMe^	127.2°	127.5°	7.7°

**Table 2:** Distribution of the CoGoMe^ according to the ANPg^ and the SN^GoGn. Differences in CoGoMe^ among individuals with different ANPg^ and SN^GoGn were estimated as appropriate using one-way ANOVA. Bold text indicates statistically significant differences.

Variables	Groups	N	Mean	Sd	P50	P25	P75	ANOVA
ANPg^								
	Class III (≤−1°)	32	128.59°	7.8°	129.2°	123.65°	133.5°	*F*(2, 287) = 0.58, P = 0.56
	Class I (−1°< x <5°)	196	127.09°	7.8°	127.4°	122.45°	132.85°	
	Class II (≥5°)	62	126.9°	7.2°	125.9°	121.8°	130.8°	
SN^GoGn								
	Hypodivergent (≤27°)	60	120.1°	6.63°	120.4°	102.5°	134°	***F*** **(2, 287) = 77.04, P < 0.001**
	Normodivergent (27°< x <37°)	166	127.1°	6.11°	127.1°	110.2°	143.8°	
	Hyperdivergent (≥37°)	64	134.02°	6.18°	133.7°	121.7°	156.5°	

**Table 3:** Distribution of the CoGoMe^ according to the ANPg^ and the SN^GoGn. Differences in CoGoMe^ among individuals with different ANPg^ and SN^GoGn were estimated as appropriate using one-way ANOVA. Bold text indicates statistically significant associations.

	Models	B	CI 95%	P
1	CoGoMe^/Aged younger than 17 years (N = 210)	−0.6	−1.05, −0.12	**0.014**
2	CoGoMe^/Aged 17 years and older (N = 80)	0.004	−0.31, −0.32	0.98
3	SN^GoGn/CoGoMe^*Age (N = 290)	0.6	0.51, 0.67	**<0.001**
